# Factors and Mechanisms Affecting Seasonal Changes in the Prevalence of Microbiological Indicators of Water Quality and Nutrient Concentrations in Waters of the Białka River Catchment, Southern Poland

**DOI:** 10.1007/s11270-016-2931-y

**Published:** 2016-08-05

**Authors:** Anna Lenart-Boroń, Anna A. Wolanin, Łukasz Jelonkiewicz, Mirosław Żelazny

**Affiliations:** 1 Department of Microbiology, University of Agriculture in Cracow, Mickiewicza Ave. 24/28, 30-059 Cracow, Poland; 2 Department of Hydrology, Institute of Geography and Spatial Management, Jagiellonian University in Cracow, Gronostajowa 7, 30-387 Cracow, Poland

**Keywords:** Temporal changes, Land use, Coliforms, *Escherichia coli*, Nutrients

## Abstract

This 3-year study was aimed to understand the factors and mechanisms that cause the temporal changes in the concentration of microbiological indicators of water quality and nutrient concentration in selected sites of the Białka river catchment (southern Poland) situated in direct vicinity of the largest ski station in the region. The analysis comprised 35 sampling campaigns conducted in five sites. Water temperature, pH, and electrical conductivity were measured during sampling, laboratory analyses included determination of the selected nutrients content (NH_4_, NO_3_, NO_2_, PO_4_); and the number of mesophilic and psychrophilic bacteria, coliforms, fecal coliforms, and *Escherichia coli*. Based on the cluster analysis, the collected samples were grouped into three to four groups, depending on the most characteristic features. Seasonal variation was evident, showing the predominance of either anthropogenic or natural-environment factors, depending on the considered season. On the other hand, principal component analysis revealed clear effect of various forms of land use in different sites.

## Introduction

Water is one of the natural resources, essential for all types of human activity; therefore, preserving its quality and condition has always been very important. Water has great impact on the surrounding environments and can affect both the landscape and the land use (Bowden et al. 2015). The quality of water—its chemical parameters as well as the content of different groups of microorganisms—is one of the most important factors that affects suitability of water for its use in various aspects. Broadly understood, water quality affects health and safety of its users, plant and animal production, economic production, economic development in both production and non-production sectors, and finally, the condition of natural environment. Another important aspect is that Poland is among countries with the lowest freshwater resources per inhabitant in the European Union and Poland, next to the Czech Republic, Cyprus, and Malta, is one of the countries which experience “water stress,” i.e., its annual water resources drop below 1700 m^3^ per inhabitant (Eurostat 2015). Even though drinking water supply is based on groundwater, which is characterized by much higher quality than surface water, the latter represents as much as 85 % of Polish water resources; therefore, it is used as a main source of water supply for the Polish economy (Myszograj and Sadecka 2012). This is the reason why ensuring best possible quality of surface water becomes on the one hand the most important, but on the other, the more and more challenging task.

There are numerous, natural, and anthropogenic factors that can affect the physicochemical parameters and microbiological quality of surface water. Among the natural factors, one can mention geological structure, seasonal differences in runoff volumes, weather conditions or water levels, as well as land cover and vegetation cycle (Bartram and Ballance 1996). Anthropogenic factors include the type of land use (Bartley and Speirs 2010; Bowden et al. 2015), which can affect point and non-point sources of pollution (USEPA 2015). Land use is seen as the primary factor responsible for changes in sediment and nutrient delivery to water bodies (Bartley and Speirs 2010). Studies have shown that information on the condition of the land can greatly improve the modeling results; therefore, water quality data should be collected together with land condition information in order to help differentiate between the natural changes in water quality trends with changes resulting from land management (Bartley and Speirs 2010). Also non-point sources of pollution may form a mixture of natural and anthropogenic factors, such as in the case of rainfall and snowmelt runoff carrying away the pollutants from the ground finally depositing them into waters (USEPA 2015). On the other hand, point sources of pollution are mainly the effluents from municipal and industrial wastewater treatment plants (Nnane et al. 2011). Also, the concentration of microorganisms in water, particularly those related to fecal contamination, may depend on their sources, including wildlife, farming, and various types of human activity (Meays et al. 2006) such as uncontrolled sewage discharge which may introduce large numbers of fecal bacteria, including those of pathogenic species (Lenart-Boroń et al. 2016).

Different landscapes and types of land use may prevail in various sites of a single catchment. It is therefore important to conduct studies in order to better understand the mechanisms and factors affecting changes in the quality of surface water in different types of landscapes and/or land use. This may help in developing field protocols describing the sampling frequency, number, and density of sites located throughout a catchment and to verify statistical methods used to analyze the results of studies conducted in a watershed.

The primary objective of this 3-year study was to track the seasonal changes in the concentration of microbiological indicators of water quality and the concentration of nutrients in the selected sites of the Białka river catchment (southern Poland), situated in close proximity to one of the largest ski resorts in the region. Another objective was to use cluster analysis and principal component analysis to establish the most possible factors affecting the chemical and microbiological quality of water in the studied sites and its seasonal changes.

## Material and Methods

### Study Site and Sampling Strategy

**Fig. 1:**
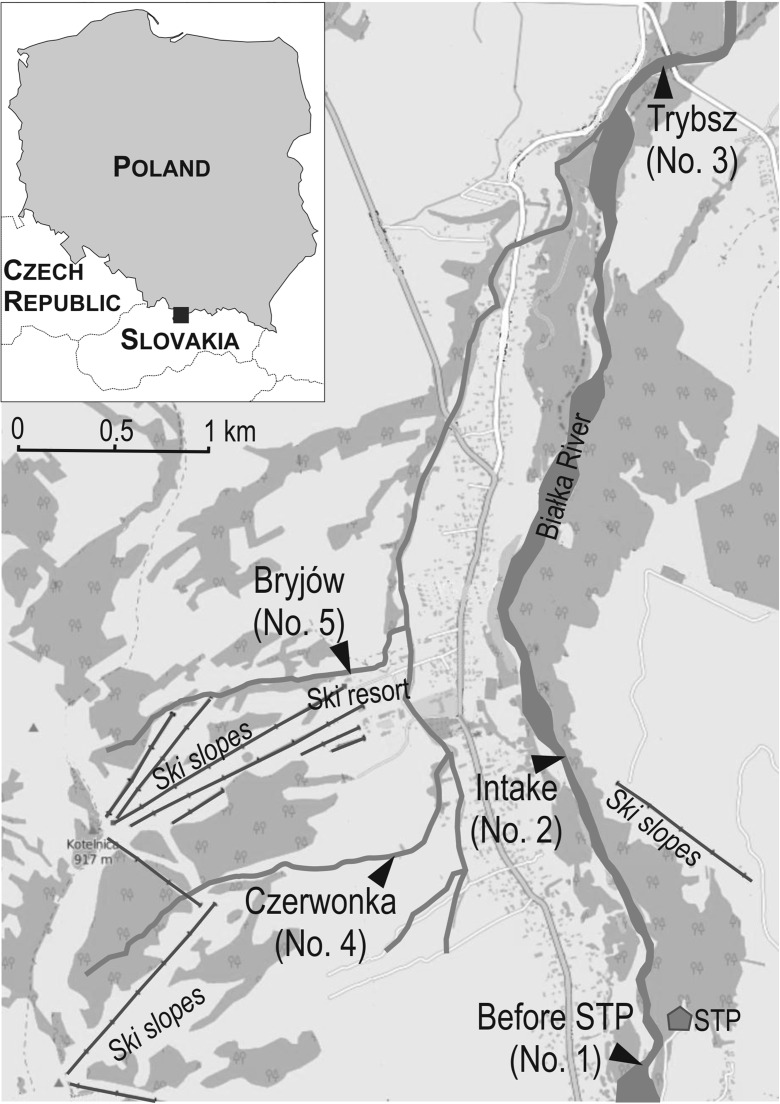
Sampling sites

The study site covers the Białka river valley (Podhale region, southern Poland), from the Tatra National Park to the village of Trybsz, situated c.a. 15 km from the mouth of the river to the Czorsztyńskie Lake (Fig. 1). The majority of the Białka catchment is covered by two protected areas—Tatra National Park and Natura 2000 network.

Five sampling sites were selected for the analysis (Fig. 1)—three of them situated along the Białka river and two on the tributaries of Białka. The first sampling site is located before the municipal sewage treatment plant (no. 1), no. 2 is located at the intake of water for artificial snowing of the slopes of the Kotelnica Białczańska ski resort, and the third one at the border of the municipality—Trybsz village (no. 3). The two tributaries include the Czerwonka stream (no. 4) and Bryjów (no. 5).

Water samples were collected over a period of 3 years in 35 sampling campaigns, conducted every month starting from January 2013 to October 2015. Each sample was collected into two bottles—1000 ml autoclaved polypropylene bottles for microbiological analyses and 500 ml polyethylene bottles for chemical assessments. Water temperature, electrical conductivity (EC_25 °C_), and pH were measured onsite during sampling using a Pro 2030 Multimeter handheld (YSI, US).

### Laboratory Analyses

The numbers of total coliforms (purple red colonies with metallic sheen on Endo agar, incubation at 37 °C, 48 h), thermotolerant coliforms (purple red colonies with metallic sheen on Endo agar, incubation at 44 °C, 48 h) and *Escherichia coli* (blue-green colonies on TBX agar, incubation at 37 °C—total; and 44 °C—thermotolerant/fecal, 48 h) were determined using a membrane filtration method. Enumeration of mesophilic bacteria (trypticase soy agar, 37 °C, 48 h) and psychrophilic bacteria (trypticase soy agar, 22 °C, 72 h) was conducted using a serial dilutions method. After incubation, grown colonies were counted and expressed as colony-forming units per 100 ml in membrane filtration method and per 1 ml in a serial dilutions method (CFU/100 ml and CFU/ml).

Chemical composition of water was determined in the laboratory of the Institute of Geography and Spatial Management, Jagiellonian University in Kraków. After filtration of water with a 0.45-μm PTFE syringe filter, the chemical composition of water was determined by ion chromatography using two chromatographs DIONEX ICS-2000 and an autosampler AS-40. The chromatographic system composed of anionic and cationic modules allows the simultaneous separation and determination of the following ions in water: NH_4_, NO_3_, NO_2_, PO_4_.

### Statistical Analysis

Cluster analysis (CA) and principal component analysis (PCA) were used in order to determine the relationship between microbiological indicators and water quality parameters as well as to explain natural and anthropogenic processes that affect changes in these characteristics. CA and PCA were performed for each of the studied sites based on the following variables: total coliforms, fecal coliforms, *E. coli*, mesophilic bacteria and psychrophilic bacteria together with the temperature of water, pH, EC_25 °C_, and the concentration of NH_4_, NO_3_, NO_2_, and PO_4_ ions. CA is one of the most commonly used methods of water quality data classification and allows to combine water samples into groups with most similar characteristics. Euclidean distance was adopted as a measure of similarity. Ward’s agglomerative clustering, which involves estimating the distance between clusters by the analysis of variance, was adopted as a grouping method. The PCA method can be used to extract key information from microbiological and physicochemical data sets and in order to identify the factors influencing the quality of water. Two most important factors for each measurement point were selected for the interpretation.

## Results and Discussion

Table 1 presents basic microbiological indicators of water quality and physicochemical characteristics of water in the examined sites together with the coefficient of variation (CV) of these parameters. Microbiological parameters are characterized by significant variations between individual sites. At the same time, we can observe very large diversity over the study period, expressed by the CV. The analyzed water samples are characterized by mean conductivity ranging from 214.9 to 389.9 μS cm^−1^ and small concentrations of nitrogen and phosphorus compounds.

**Table 1:** Basic statistics of microbiological and physicochemical parameters of waters at individual sampling sites

Site	Param.	Coliforms	*E. coli*	Fecal coliforms	Fecal *E. coli*	Mesoph. bacteria	Psychroph. bacteria	*T*	pH	EC_25 °C_	NH_4_	NO_3_	NO_2_	PO_4_
		CFU 100 mL^−1^				CFU mL^−1^		°C	–	μS cm^−1^	mg L^−1^			
Before STP	Mean	5577	2753	1751	579	458	1777	6.3	7.9	214.9	0.020	2.664	0.020	0.011
	Q25 %	21	0	3	0	23	70	2.5	7.9	180.9	0.006	1.906	0.001	0.003
	Q75 %	3120	150	1010	160	555	1868	9.5	8.1	252.0	0.028	2.984	0.001	0.003
	CV [%]	308.9	512.5	249.6	279.5	159.9	252.0	76.5	3.9	20.8	93.8	36.8	492.0	253.7
Trybsz	Mean	6007	3340	4334	1690	2738	12204	6.2	8.0	233.5	0.110	3.027	0.036	0.064
	Q25 %	139	1	26	0	131	273	1.5	7.9	198.3	0.007	2.112	0.001	0.003
	Q75 %	4405	1200	4565	1535	780	3765	8.7	8.2	268.5	0.130	3.943	0.032	0.071
	CV [%]	213.9	240.4	205.5	259.3	238.6	449.3	81.7	4.7	20.6	141.0	37.6	191.4	201.2
Intake	Mean	5977	1646	2809	2047	1869	4218	6.0	8.0	221.7	0.083	2.733	0.018	0.058
	Q25 %	230	0	3	0	142	264	2.1	7.9	181.2	0.023	2.037	0.001	0.003
	Q75 %	5280	1068	835	330	1600	3338	8.6	8.2	264.3	0.089	3.462	0.008	0.060
	CV [%]	241.4	241.0	242.7	278.6	183.2	217.6	78.9	3.8	22.1	130.8	40.5	322.3	193.0
Bryjów	Mean	2599	389	2189	347	1456	5161	6.4	8.0	373.8	0.244	3.716	0.025	0.041
	Q25 %	11	0	4	0	30	130	3.7	7.8	328.0	0.010	1.939	0.001	0.003
	Q75 %	228	75	115	41	378	1173	9.2	8.1	424.2	0.043	4.367	0.001	0.054
	CV [%]	458.0	268.1	459.6	279.3	207.5	288.9	60.9	2.9	18.4	387.1	90.5	285.0	229.0
Czerwonka	Mean	4300	393	159	68	10855	5503	5.8	8.0	389.9	0.595	2.053	0.021	0.306
	Q25 %	16	0	8	0	37	400	2.9	7.8	329.0	0.000	1.254	0.001	0.003
	Q75 %	292	74	194	51	1790	3600	9.3	8.2	418.0	0.119	2.689	0.001	0.009
	CV [%]	534.4	462.2	155.8	237.1	341.4	191.8	71.7	2.7	23.5	348.1	49.2	228.2	352.6

**Fig. 2:**
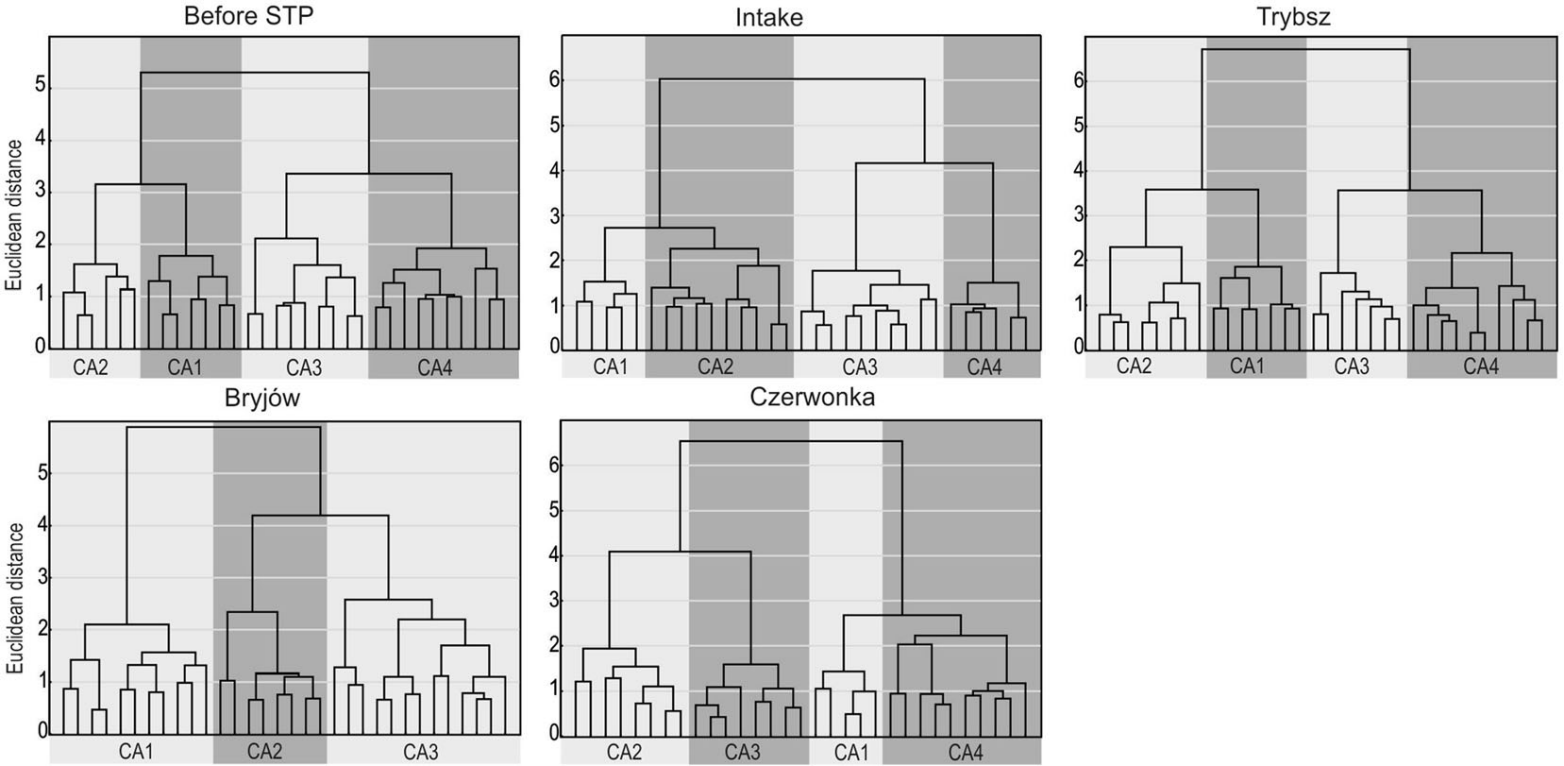
Dendrograms based on Ward’s cluster analysis of streamwaters

The cluster analysis allowed to separate from three to four groups. The result of grouping by the CA method is presented in Fig 2, while Table 2 shows the median values for physical and chemical parameters of water for individual groups designated by cluster analysis. At the sampling sites, before STP, Trybsz, and Czerwonka, cluster analysis allowed for the separation of four groups with similar microbiological and chemical relationships.

**Table 2:** Median values for microbiological and physicochemical parameters of groups designated using CA for the analyzed sites

Site	Group	Coliforms	*E. coli*	Fecal coliforms	Fecal *E. coli*	Mesoph. bacteria	Psychroph. bacteria	*T*	EC_25 °C_	pH	NH_4_	NO_3_	NO_2_	PO_4_
		CFU 100 mL^−1^				CFU mL^−1^		°C	μS cm^−1^	–	mg L^−1^			
Before STP	CA2	2118	383	4173	860	1090	1450	9.5	7.8	176.1	0.011	1.906	0.001	0.003
	CA1	4000	1130	820	56	85	980	4.6	8.0	268.9	0.026	3.002	0.001	0.003
	CA3	29	0	9	0	120	270	11.7	8.0	186.7	0.006	1.826	0.001	0.003
	CA4	46	0	7	0	74	50	1.9	8.0	236.7	0.019	2.684	0.001	0.003
Intake	CA1	11,000	7200	13,450	7500	4900	5200	4.3	7.8	202.6	0.091	2.959	0.049	0.055
	CA2	1335	310	541	37	553	868	4.2	8.0	246.6	0.040	3.196	0.001	0.049
	CA3	815	20	23	0	214	615	9.1	8.0	186.8	0.033	1.807	0.001	0.003
	CA4	81	0	2	0	868	645	2.1	8.2	267.7	0.055	3.173	0.001	0.003
Trybsz	CA2	2900	1200	4660	1343	780	2600	8.2	7.9	175.4	0.019	1.978	0.008	0.003
	CA1	10,000	4750	5410	1785	395	3850	3.9	7.9	252.3	0.055	4.113	0.014	0.073
	CA3	110	1	20	0	400	285	10.6	8.0	213.6	0.004	2.344	0.001	0.003
	CA4	185	7	85	0	270	400	0.8	8.1	268.5	0.130	3.725	0.001	0.003
Bryjów Stream	CA1	670	204	510	50	137	765	9.5	8.0	410.5	0.0017	2.2900	0.0008	0.003
	CA2	18	0	1	0	245	605	5.6	8.1	429.8	0.0200	2.0236	0.0008	0.003
	CA3	15	0	16	1	39	400	3.8	8.0	309.1	0.0396	4.6035	0.0008	0.003
Czerwonka Stream	CA2	288	74	247	74	2275	529	9.3	7.8	385.4	0.0051	1.5024	0.0008	0.003
	CA3	16	0	8	2	23	400	9.2	8.2	401.5	0.0011	1.2329	0.0008	0.003
	CA1	410	170	113	22	1330	4240	1.1	8.1	400.0	0.0806	2.7052	0.0013	0.008
	CA4	52	0	30	0	320	1208	3.1	8.0	408.2	0.2356	2.7378	0.0010	0.0074

Group 1 clustered waters with very high concentration of bacteria, low temperature, high EC_25 °C_ values, and the concentrations of nitrogen and phosphorus compounds. Such characteristics of water were observed in winter months, during the periods of highest tourist traffic (winter holidays). Since ski infrastructure and accommodation base are very well developed in the study area, water demand for municipal purposes and for snowing ski slopes is high, as well as a large amount of wastewater is produced. Unfortunately, only 49.8 % of local tenants is connected to the sewage treatment plant (Central Statistical Office 2013). There are also numerous discharge sites of untreated sewage that enter local rivers and streams. For these reasons, the quality of water deteriorates in the investigated sites.

Group 2 clusters waters with high content of bacteria, low EC_25 °C_ values, and low concentrations of NH_4_, NO_3_, NO_2_, and PO_4_. Such characteristics are typical of spring months during snowmelt periods. At this period, water is diluted by melting snow, hence low EC_25 °C_ values and low ion content (Ahearn et al. 2004). At the same time, bacteria are being washed out from the soil and residuals, hence their high numbers in water.

Group 3 is characterized by small numbers of bacteria, low values of nitrogen and phosphorus, and high temperature of water. Such features occur mostly in late spring and summer. Assimilation of nutrients, which occurs during vegetation, decreases their concentration in stream waters (Campbell et al. 2000; Clark et al. 2004). At the same time, water contains lower amounts of bacteria, since soil leaching is less intense. In the considered region, the number of tourists in summer is significantly lower than in winter, and therefore, the amount of sewage discharged into stream waters is smaller.

Group 4 is characterized by high EC_25 °C_ values, low water temperature, small numbers of bacteria, and increased concentrations of nitrogen and phosphorus compounds. Such relationships between the microbiological and physicochemical characteristics are observed mostly in autumn and early winter. Slightly higher concentrations of N and P compounds result from limited assimilation by plants, since there is no vegetation at that time.

CA allowed to determine three water clusters for the Bryjów stream. Cluster 1 is characterized by high numbers of bacteria, high temperature, and low contents of nitrogen and phosphorus. This group comprises samples collected in the period of late spring and in summer. At that time, the period of snowmelt from artificially snowed slopes ends in this stream’s catchment and bacteria are being washed out from the soil cover. Low concentrations of nitrogen and phosphorus are due to the fact that most of their compounds were leached out at the beginning of snowmelt, because as demonstrated by Johannessen and Henriksen (1978), up to 80 % of ions contained in snow is released in the initial stage of melting. In addition, the process of assimilation of these compounds by plants occurs at that time. In group 2, the stream waters are characterized by high conductivity, small numbers of bacteria, and low concentrations of N and P compounds. Such samples are collected usually in late autumn and early spring. Finally, group 3 is characterized by small number of bacteria, low conductivity but high concentrations of nitrogen compounds. This is the time (late winter, early spring) when snowmelt begins on the ski slopes and ions such as NO_3_ and NH_4_ are first released from snow in the process of melt fractionation and preferential elution (Brimblecombe et al. 1985).

Based on the cluster analysis, four groups were distinguished at the site intake. Group 1 contains the highest number of bacteria and high concentrations of nitrogen and phosphorus compounds. In contrast, pH of water is low. This group comprised samples collected in winter, during the most intense tourist traffic. Probably, the sewage treatment plant, which is situated at a short distance from this sampling site, caused such deterioration of water quality. With so much tourist traffic, the treatment plant is unable to purify such large amount of sewage and discharges untreated wastewater into the river (Lenart-Boroń et al. 2016). Group 2 comprises waters with relatively high number of bacteria, high conductivity, and quite high concentrations of NH_4_, NO_3_, and PO_4_ ions. Such samples of water were collected usually at the end of winter period, when the tourist traffic is not as intense and the sewage treatment plant has less problems with wastewater purification. Group 3 is characterized by small number of bacteria, low conductivity, high temperature of water, and very low concentrations of nitrogen and phosphorus compounds. Such samples were collected in spring and summer. Low N and P concentrations indicate their assimilation by plants during the growing season. Group 4 clusters waters with very small numbers of bacteria, low temperature, high conductivity, and slightly higher contents of NH_4_ and NO_3_. Such samples were collected in the beginning of winter, before the tourist season. This is why low prevalence of bacteria is found at that time. In contrast, the ending of the growing season causes higher concentrations of nitrogen compounds, because their assimilation no longer occurs.

**Fig. 3:**
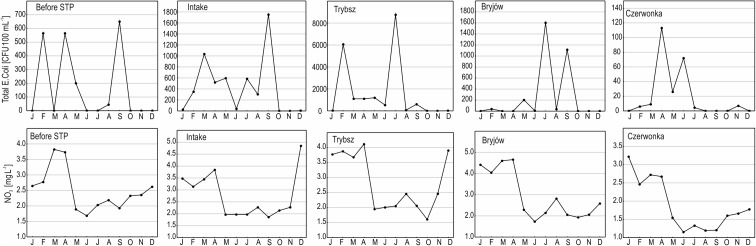
Seasonal changes in the number of total *E. coli* and the concentration of NO_3_ in the studied sampling sites

Figure 3 presents seasonal variation in the number of *E. coli* and the concentration of NO_3_ in the studied sampling sites. These characteristics confirm the results obtained from the cluster analysis. The increased number of bacteria in the winter months, during heavy tourist traffic, and during spring thaw is evident in most of the sites. On the other hand, the concentrations of NO_3_ are the highest in winter and in early spring (human impact and release from melting snow), but they are low in the summer when these compounds are assimilated by plants.

Based on microbiological indicators and physicochemical parameters of water in the studied sites, principal component analysis (PCA) allowed to designate two main factors (Table 3), which explain in total from 40.7 % (before STP) to 59.7 % of variance (intake). Factor 1 (PC1) explains from 23.5 to 36.8 % of variance, while factor 2 (PC2)—from 17.3 to 23.8 %.

**Table 3:** Factor loadings for microbiological and physicochemical parameters of streamwater

Parameter	Unit	Before STP		Intake		Trybsz		Bryjów Stream		Czerwonka Stream	
		PC1	PC2	PC1	PC2	PC1	PC2	PC1	PC2	PC1	PC2
Total coliforms	CFU 100 mL^−1^	0.45	0.34	*−0.81*	−0.01	*−0.62*	−0.37	−0.29	−0.42	0.38	0.51
Total *E. coli*		0.41	0.28	*−0.85*	−0.13	*−0.85*	−0.35	*−0.74*	0.50	0.39	0.55
Fecal coliforms		−0.48	0.54	*−0.94*	−0.09	*−0.66*	−0.39	−0.30	−0.42	0.22	0.41
Fecal *E. coli*		−0.48	0.45	*−0.96*	−0.06	*−0.82*	−0.33	*−0.74*	0.51	0.03	0.40
Mesophilic bacteria		−0.02	−0.54	−0.52	−0.56	0.10	−0.43	−0.58	0.30	0.15	0.53
Psychrophilic bacteria		0.15	−0.16	−0.16	−0.33	−0.40	−0.59	0.23	0.49	0.06	0.49
*T*	°C	*−0.67*	0.30	−0.15	*0.80*	0.33	*−0.72*	*−0.62*	−0.20	0.11	−0.59
pH	–	0.29	*0.70*	*0.81*	−0.20	−0.22	0.33	0.31	−0.39	0.29	−0.49
EC	μS cm^−1^	*0.89*	0.14	0.38	*−0.81*	−0.44	*0.79*	−0.53	−0.57	*−0.79*	−0.28
NH_4_	mg L^−1^	0.40	0.35	0.50	−0.45	*−0.62*	0.55	0.53	−0.26	*−0.88*	0.24
NO_3_		*0.78*	−0.24	−0.05	*−0.88*	−0.23	*0.63*	0.52	0.50	0.07	*0.70*
NO_2_		−0.21	*−0.68*	−0.21	0.09	−0.50	−0.09	0.44	0.29	*−0.66*	0.42
PO_4_		0.25	−0.04	−0.43	−0.44	−0.48	0.30	0.05	0.40	*−0.91*	0.22
Accounted variance (%)	23.5	17.3	36.8	22.9	28.1	23.8	24.5	17.5	24.1	21.8	

Loadings ≥0.60 are in italics

At the sampling site before STP, factor 1 most clearly demonstrates the negative relation between EC_25 °C_, NO_3_, and temperature. This means that the higher NO_3_ concentration and conductivity, the lower the water temperature. This factor shows the effect of climatic conditions and the growing period on the natural seasonal variability of nitrate concentration and conductivity. On the other hand, factor 2 demonstrates the negative relationship between the content of fecal coliforms and water pH, and mesophilic bacteria coupled with the concentration of NO_2_. Thus, the higher pH of water, the higher content of fecal coliforms but lower content of mesophilic bacteria and lower NO_2_ concentrations. pH of water may increase with inflow of certain wastewater contaminants, which may be also evidenced by the positive correlation with the number of fecal coliforms, while on the other hand, increased pH may inhibit the proliferation of mesophilic bacteria, which may occur in the examined sampling site as a result of surface runoff (Chomutowska 2009).

At the intake, factor 1 demonstrates significant negative relationship between the content of total coliforms, *E. coli*, fecal coliforms, fecal *E. coli*, mesophilic bacteria, and water pH. This indicates that the lower pH of water, the higher content of these bacteria in water. This phenomenon may reflect the seasonal changes that occur at this site, i.e., pH of water may decrease with surface runoff from the areas covered by coniferous forests (Nisbet and Evans 2014). Runoff water may also contain all mentioned groups of bacteria, particularly high numbers of total coliforms, total *E. coli*, and mesophilic bacteria, but fecal bacteria can also occur in large numbers. On the other hand, factor 2 shows a negative relationship between mesophilic bacteria, EC_25 °C_, NO_3_, and water temperature; thus, the lower temperature of water, the higher EC_25 °C_ and NO_3_ and more mesophilic bacteria. This relationship may also reflect the temporal changes occurring in the considered region, i.e., lower temperature of water being detected from November to March, which is also the period of increased tourist traffic. This results in increased amount of sewage produced, of which only a part is disposed to the treatment plant while many households discharge untreated sewage (containing also wide variety of bacteria) directly to the river (Lenart-Boroń et al. 2016).

At the sampling site Trybsz, factor 1 shows positive correlation between the number of coliforms, *E. coli*, fecal coliforms, fecal *E. coli*, and the concentrations of NH_4_ and NO_2_. This means that the higher content of coliforms and *E. coli*, the higher concentration of ammonium and nitrite, which clearly evidences the impact of anthropogenic pressure on the quality of water in the studied river. On the other hand, factor 2 shows negative relation between psychrophilic bacteria and temperature, and EC_25 °C_, NH_4_, NO_3_. Therefore, the higher temperature of water, the more psychrophilic bacteria, but smaller concentrations of NH_4_ and NO_3_. Variations in water temperature at this sampling site are mainly related to seasonal changes—water temperature increases in spring and drops in late autumn. Therefore, the period of higher water temperature is the period when water at this site is much cleaner than in winter (Lenart-Boroń et al. 2016), which may result in the observed higher prevalence of psychrophilic bacteria, whose numbers dominate over mesophilic bacteria. In most cases, the group of psychrophilic bacteria consists non-pathogenic species (Donderski and Wilk 2002).

At the Bryjów spring, PC1 shows the negative relationship between total *E. coli*, fecal *E. coli*, mesophilic bacteria, water temperature, EC_25 °C_, and NH_4_ and NO_3_. This means that the higher water temperature and greater conductivity, the greater the number of mentioned bacteria but smaller concentrations of NH_4_ and NO_3_. This relationship may result from temporal changes in the usage of the area surrounding the considered sampling site. Greater numbers of microorganisms coupled with increased temperature and conductivity of water may be the effect of surface runoff after snowmelt, coupled with bacteria derived from feces of sheep grazing on the slopes from which water supplies the considered sampling site. This also coincides with the end of the ski season (spring), which means that seasonally operating restaurants which produce and discharge sewage into the stream are closed, hence smaller concentration of chemical indicators of fecal contamination. On the other hand, PC2 explains a negative relationship between EC_25 °C_ and total number of *E. coli*, fecal *E. coli*, and NO_3_. This means that the higher the conductivity, the lower the number of bacteria and concentration of NO_3_. Such relationship can be observed in autumn, when nitrates are still assimilated by plants while the human impact is small, which is associated with smaller numbers of total *E. coli* and fecal *E. coli*.

At the sampling site Czerwonka, factor 1 indicates the positive relationship between EC_25 °C_ and NH_4_, NO_2_, and PO_4_ ions. PC2 explains the negative relationship between coliforms, *E. coli*, mesophilic bacteria and NO_3_, and water temperature. Thus, the higher temperature of water, the lower concentrations of NO_3_ and less total coliforms, *E. coli*, and mesophilic bacteria. This is another evidence for seasonal/temporal changes occurring in the studied area, as water temperature at this site increases in spring when the ski season ends; therefore, the potential contamination sources for this sampling site, i.e., bars that operate only seasonally and discharge their sewage into the stream, are closed for the remaining part of the year.

## Conclusions

This 3-year study showed evident seasonal variation in both nutrients and bacterial indicators of water quality in the considered sampling sites. Such significant temporal changes in the analyzed parameters result from the fact that various factors prevail in different seasons of the year coupled with the diversity of land use in the considered area.

The applied statistical tests allowed for reliable determination of factors affecting the observed changes in water quality and the mechanisms affecting variable importance of these factors throughout the year.

The cluster analysis indicated that depending on the season of the year, the prevailing factors are either related to anthropogenic pressure, such as changing intensity of tourist traffic coupled with changing efficiency of the local STP, or they are strictly environmental (i.e., snowmelt, nutrient assimilation by plants or soil leaching). On the other hand, principal component analysis indicated the effect of land use which varies both between the sampling sites and—in some locations—throughout the year.

Precise identification of the sources of water contamination can help develop management plans in order to reduce the contamination and its effect on the watershed. This study indicates the importance of conducting long-term observations, which at the same time need to be coupled with complex understanding of local conditions and their detailed analysis.
